# Editorial

**Published:** 1899-08

**Authors:** 


### HOMŒOPATHIC PHYSICIAN,

A MONTHLY JOURNAL OF
HOMŒOPATHIC MATERIA MEDICA AND CLINICAL MEDICINE.

If our school ever gives up the strict inductive method of Hahnemann, we are lost, and deserve only to be mentioned as a caricature in the history of medicine."—CONSTANTINE HERING.

Vol. XIX. AUGUST, 1899. No. 8.

### EDITORIAL.

THE INTERNATIONAL HAHNEMANNIAN ASSOCIATION MEETING was a great success, being both instructive and interesting, notwithstanding the few members and the shortness of the session. This is the more gratifying since for two months preceding the time of meeting an underhand attempt of the most determined and concerted character had been made to nullify the meeting and break up the I. H. A. completely.

Although the utmost secrecy was practiced, yet accidental and unintentional intimations of this malignant scheme had been communicated to the President several weeks before the meeting, leaving him with the firm conviction that when he would go to the Convention he should have the unique experience of presiding over nothing but empty chairs. Notwithstanding, he resolved to do his duty, and intended if only *one* member appeared on the scene he would nevertheless hold a meeting with the aid of that one member and save the Association, if possible, from this dastardly attempt to wreck it. He was successful beyond his best hopes, and when the hour of assembling arrived, he was pleased to see twelve members present, who were superior to the influences of this ridiculous intrigue.

A harmonious and instructive series of sessions was the result, absolutely free of the vexatious and underhand intrigues that have threatened the continuance of so many conventions in the past. Attention not being divided with these small plots, it was instead concentrated on the legitimate work of the Society, and hence the success already recorded. Another set of officers who would carry on the work for another year was elected, and thus the Association was saved from the threatened destruction.

A short summary of the proceedings together with the list of members in actual attendance will be found in this number. The President's address has been already published in the July number.

